# Supplementary material for: Idiopathic Pulmonary Arterial Hypertension in Paediatrics Represents Still a Serious Challenge: A Case Series Study
Source: Children (Basel). 2023 Mar 6;10(3):518. doi: 10.3390/children10030518 (PMC10047811; doi:10.3390/children10030518)
Supplement: Supplementary file 1 [file children-10-00518-s001.zip › children-2139905-supplementary.pdf]

Table S1. Clinical, echocardiographic, right heart catheterisation features, and outcome of the idiopathic pulmonary hypertension patients who have been studied

Patient	Gender	Age at first diagnosis	NYHA functional class at diagnosis	Age at last follow-up	Saturation on room air	Therapy	Echo TAPSE	Echo RVSP	RHC mPAP	RHC mRAP	RHC CI	RHC PVRi	RHC SVO2	Outcome
1	F	1	II	1	87	Sildenafil, Bosentan	9	90	62	10	2	22	60	On transplant list
2	M	6	II	16	93	Sildenafil, Bosentan, Treprostinil	12	85	107	17	3.6	18.8	69	On transplant list
3	F	4	II	13	80	Sildenafil, Ambrisentan, Treprostinil	13	130	80	13	5.3	24	66	On transplant list
4	F	11	IV	16	88	Sildenafil, Bosentan, Epoprostenol (upfront therapy)	12	90	82	9	2	23	41	Much improved with therapy (currently NYHA I). Epoprostenol switched to Treprostinil
5	F	5	II	9	93	Sildenafil, Bosentan, Epoprostenol	9	100	41	8	5.2	8.2	84	Lung transplant
6	M	17	III	17	92	No therapy. Addressed straight to lungs transplant	16	125	57	16	2.8	18.8	52	Death while waiting for lung transplant
7	M	11	III	13	90	Sildenafil, Bosentan, Iloprost	11	110	51	10	3.7	9.3	76	Death while waiting for lung transplant
8	M	0.1	III	15	97	Sildenafil, Macitentan	22	75	48	9	2.6	10.6	67	Small PDA + atrial septostomy + atrial stenting
9	F	0.1	IV	16	93	Sildenafil, Bosentan, Epoprostenol	17	120	50	7	N/A	20	56	PDA stenting (reverse Potts shunt)
10	F	2	III	8	95	Sildenafil, Bosentan, Epoprostenol	N/A	85	40	8	N/A	8.7	65	Transplant assessment

Legends: M: male; F: female; NYHA: New York Heart Association; TAPSE: tricuspid annular plane systolic excursion; N/A: not acquired; RVSP: right ventricular systolic pressure; RHC: right heart catheterisation; mPAP: mean pulmonary arterial pressure; mRAP: mean right atrial pressure; CI: cardiac index; PVRi: pulmonary vascular resistance; SVO_2: mixed venous oxygen saturation; PDA: patent ductus arteriosus.
